# EasyFlyTracker: A Simple Video Tracking Python Package for Analyzing Adult *Drosophila* Locomotor and Sleep Activity to Facilitate Revealing the Effect of Psychiatric Drugs

**DOI:** 10.3389/fnbeh.2021.809665

**Published:** 2022-02-10

**Authors:** Susu Qu, Qingjie Zhu, Han Zhou, Yuan Gao, Yi Wei, Yuan Ma, Zhicheng Wang, Xueting Sun, Lei Zhang, Quanjun Yang, Lei Kong, Li Zhang

**Affiliations:** ^1^Academy for Advanced Interdisciplinary Studies, Peking University, Beijing, China; ^2^Chinese Institute for Brain Research, Beijing, China; ^3^Beijing Advanced Innovation Center for Big Data-Based Precision Medicine, Capital Medical University, Beijing, China; ^4^Department of Pharmacy, Shanghai Jiao Tong University Affiliated Sixth People’s Hospital, Shanghai, China; ^5^Center for Bioinformatics, State Key Laboratory of Protein and Plant Gene Research, School of Life Sciences, Peking University, Beijing, China

**Keywords:** *Drosophila*, video tracking software, psychiatric drug, locomotor activity, hyperactivity-like behavior

## Abstract

The mechanism of psychiatric drugs (stimulant and non-stimulant) is still unclear. Precision medication of psychiatric disorders faces challenges in pharmacogenetics and pharmacodynamics research due to difficulties in recruiting human subjects because of possibility of substance abuse and relatively small sample sizes. *Drosophila* is a powerful animal model for large-scale studies of drug effects based on the precise quantification of behavior. However, a user-friendly system for high-throughput simultaneous tracking and analysis of drug-treated individual adult flies is still lacking. It is critical to quickly setup a working environment including both the hardware and software at a reasonable cost. Thus, we have developed EasyFlyTracker, an open-source Python package that can track single fruit fly in each arena and analyze *Drosophila* locomotor and sleep activity based on video recording to facilitate revealing the psychiatric drug effects. The current version does not support multiple fruit fly tracking. Compared with existing software, EasyFlyTracker has the advantages of low cost, easy setup and scaling, rich statistics of movement trajectories, and compatibility with different video recording systems. Also, it accepts multiple video formats such as common MP4 and AVI formats. EasyFlyTracker provides a cross-platform and user-friendly interface combining command line and graphic configurations, which allows users to intuitively understand the process of tracking and downstream analyses and automatically generates multiple files, especially plots. Users can install EasyFlyTracker, go through tutorials, and give feedback on http://easyflytracker.cibr.ac.cn. Moreover, we tested EasyFlyTracker in a study of *Drosophila melanogaster* on the hyperactivity-like behavior effects of two psychiatric drugs, methylphenidate and atomoxetine, which are two commonly used drugs treating attention-deficit/hyperactivity disorder (ADHD) in human. This software has the potential to accelerate basic research on drug effect studies with fruit flies.

## Introduction

*Drosophila* is a powerful genetic animal model for studies of complex phenotypes such as circadian rhythms, sleep, movement, and diseases (Sokolowski, 2001; Bier, 2005; Bellen et al., 2010). With lower costs and higher yields than mammalian models, *Drosophila* has contributed to revealing the genetic and neuroscientific basis of autism spectrum disorders (ASDs) (Tian et al., 2017; Coll-Tane et al., 2021), attention-deficit/hyperactivity disorder (ADHD) (van Swinderen and Brembs, 2010; van der Voet et al., 2016), and other disorders (Ries et al., 2017). In particular, large-scale studies of target genes and drug effects of the stimulants such as amphetamine, methylphenidate (MPH), and cocaine have greatly accelerated the basis of future pharmacogenomic and pharmacodynamic research (Heberlein et al., 2009; Rohde et al., 2019; Philipsen et al., 2020). Sleep and locomotor activity are crucial behaviors in the study of neurological disorders in *Drosophila*, since certain psychiatric disorders cause deficits in these behaviors. Different devices and accompanying software have been proposed for *Drosophila* sleep/locomotor tracking and downstream analyses, but they are not designed for the simultaneous independently tracking of multiple individual flies in drug effect studies. For example, pySolo (Gilestro and Cirelli, 2009), ShinyR-DAM (Cichewicz and Hirsh, 2018), and “tracker” (Donelson et al., 2012) software have been widely used, but limited to the infrared-detected *Drosophila* Activity Monitor (DAM) system (TriKinetics, Waltham, MA, United States). It records the frequency of fruit flies crossing infrared beams in a tube to study the locomotor, sleep, and circadian rhythms. The high cost of the single tube device limits its usage for high-throughput studies. Other well-known commercial tracking software, such as EthoVision XT from Noldus (Wageningen, Netherlands), is also expensive.

The Ctrax (Slawson et al., 2009) and the IowaFLI Tracker (Scaplen et al., 2019) are all camera-based software based on grouped individual tracking in the defined area. However, such group-based activity can interfere (e.g., with social behavior) with sleep/locomotor activity after drug treatment. Furthermore, some software was developed in MATLAB (Barwell et al., 2021), which is also an expansive commercial solution. In addition, it is slow when dealing with large videos using an artificial intelligence approach to track large fruit fly behaviors such as idtracker.ai (Romero-Ferrero et al., 2019).

Thus, we developed EasyFlyTracker, which uses affordable and easy-to-build equipment to track and analyze the sleep/locomotor activities of individual adult fruit flies for the study of drug effects, especially psychiatric drugs. To avoid interference of social behaviors, each arena contains only single fruit fly. EasyFlyTracker can track the activities of up to 72 individuals simultaneously with current settings and scale up to any number of individuals theoretically. After evaluating the tracking accuracy of EasyFlyTracker, we used it to track and quantify the locomotor activities of *Drosophila* treated with two commonly used psychiatric drugs such as MPH (a stimulant) and atomoxetine (ATX) (a non-stimulant) for ADHD symptoms in humans and finally identified hyperactivity-like behavior.

## Materials and Methods

Our tracking system consists of two parts, software and hardware setup, of which software (named EasyFlyTracker) development is our focus. All the hardware can be purchased directly online and installed easily and we provided product lists on our website http://easyflytracker.cibr.ac.cn/#/document. Next, for convenience of users, hardware setup is introduced first.

### Hardware Setup Requirements

**FIGURE 1:**
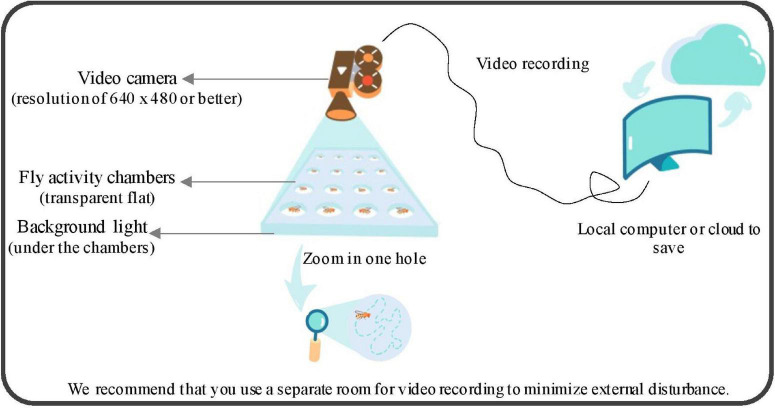
Hardware setup of video recording system. A cartoon schematic of our shooting setup, which consists of the following parts: a standard commercial video camera, a background light, a computer, and polycarbonate (PC) antistatic transparent flat chambers. The minimum size of the fruit fly body needs to be at least 4 pixels otherwise it will be treated as noise rather than a fly.

We built the customized recording environments, which are easily rebuilt and cost-effective compared with commercial equipment. The setup (cartoon diagram is shown in Figure 1) consists of the following parts: a standard commercial video camera, a background light, a computer, and polycarbonate (PC) antistatic transparent flat chambers. An example of up to 72 individuals were tracking simultaneously with our current settings (diameter of each circle is 16 mm) and it can scale up to any number and any circle size of individuals theoretically. However, as the number and circle size increase, the equipment settings should upgrade accordingly to maintain the performance of the system. The minimum size of the fruit fly body needs to be at least 4 pixels otherwise it will be treated as noise rather than a fruit fly. Active video of flies was obtained by recording the video directly above the activity areas of fly. Any camera with a resolution of 640 × 480 or better will work and we used 1,280 × 720 and 30 frames per second (fps). In addition, users need to ensure that the camera is still, the light (or infrared light) is constant, so that the background image is stable and stationary, and the background should be a clean and bright environment.

### Development of EasyFlyTracker for Locomotor and Sleep Activity Analysis

**FIGURE 2:**
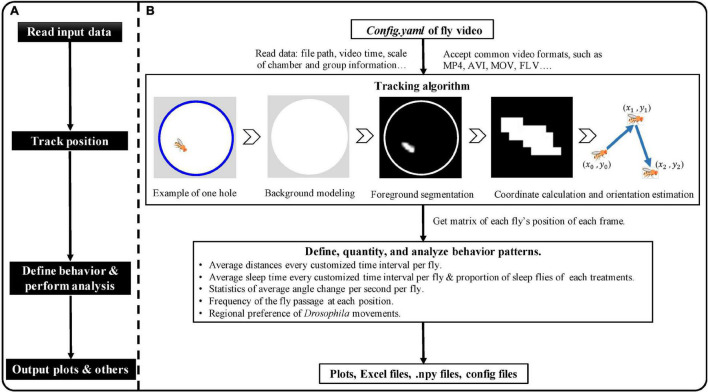
The schematic plot of EasyFlyTracker. **(A)** General flow of the software. **(B)** Details of input data, tracking algorithm, behavior definition, output, and visualization.

EasyFlyTracker is written in the open-source Python 3.6^1^ programming language and can be used to understand the tracking process, thanks to the user-friendly interface. The schematic plot of EasyFlyTracker is shown in Figure 2. General flow of the software (Figure 2A) contains read input data, track position of fly, define and analyze behavior, and output files. Details (Figure 2B) of tracking algorithm, behavior definition, outputs and visualization, and detailed information are provided below.

#### Tracking Algorithm

It calculates the trajectory of each fly (center position and orientation in each frame) after importing the stored video sequence. Sample videos,^2^ which were recorded with our aforementioned customized shooting environments, were used to develop EasyFlyTracker. Tracking is achieved through four steps (a graphical example of the tracking algorithm is shown in Figure 2B): background modeling, foreground segmentation, coordinates calculation, and orientation estimation of *Drosophila*.

##### Background Modeling

A total of 800 frames or available number of frames when it is smaller than 800 are randomly selected from video and the pixel value with the highest number of occurrences in the time dimension is kept for each pixel. The background image is obtained after traversing all the pixel points. It should be noted that a random factor is used here, which will lead to the probability of inconsistency in the results of multiple operations on the same video. However, this deviation is extremely small and belongs to the normal range.

##### Foreground Segmentation

A pixel is determined to be a foreground pixel (fruit fly) if it satisfies the following conditions: its own pixel value is less than 120 and the difference with the background pixel is greater than 70 (Piccardi, 2004). In general cases, it works very well to separate fruit flies from the background with the threshold setting as 120. However, when the background is not clean such as there are some black impurities, we need another parameter to remove the noisy pixels with the requirement that difference between the foreground and the background is greater than 70. By combining these two parameters, we can efficiently separate fruit flies from the background.

##### Coordinate Calculation of *Drosophila*

The coordinate values were calculated based on the barycenter method of the region [*connectedComponentsWithStats()*] function of OpenCV (version 4.5.2) package in Python 3.6.

##### Orientation Estimation of *Drosophila*

The minimum area boundary rectangle of the segmented fruit fly region was calculated to determine the tail-to-head orientation. We further combined the velocity direction to determine the exact location of head and tail. Due to the low resolution, we did not consider the difference between head and abdomen velocity directions as previously reported (Geurten et al., 2014).

#### Behavior Definition

Based on the trajectory matrix of each fly (center position and orientation in each frame), EasyFlyTracker quantifies behavioral patterns of locomotor and sleep activity. Average distances every 10 min per fly (10 min is commonly used in the published literature and actually users can set any customized values) are used to define locomotor activity (Rohde et al., 2019). Sleep is defined as more than 5 min complete inactivity (Shaw et al., 2000); then, statistics of average sleep time and proportion of sleeping flies of each treatment group are used to show the status of sleep activity. The treatment group information is user defined and provided in the file “group.xlsx.” The example file can be found online (see text footnote 2), which includes treatments (drugs, control, or others) and the corresponding fruit fly number. Moreover, statistics of average angle change per second per fruit fly, the frequency of the fly passage at each position, and the regional preference of *Drosophila* movements are also defined to describe the locomotor activity of fruit fly. All these statistics are provided in different formats for users.

#### Software Outputs

The software provides different outputs. The first outputs are the plots of different behaviors including the locomotor activity plot, sleep status plot, heatmap plot, angle change plot, and regional preference plot. The locomotor activity plot shows average distances of the different *Drosophila* treatment groups during different time intervals (default every 10 min). The sleep status plot displays the statistics of sleep fly (default every 30 min). The heatmap plots show the relative frequency of the fly passage at each position and both the frequency per flies and grouped heatmaps are provided. Sleep intervals can be removed from the heatmap plots with the “heatmap_remove_sleep” parameter defined in the “*config.yaml*” file. The angle change plots show the statistics of average angle change per second per fruit fly and the regional preference of *Drosophila* movements more visually shows the regional bias of *Drosophila* movement. About the details of visualization parameters, please refer to our Supplementary Material. The second outputs are Excel files, which provide analysis results of different behaviors among the different groups and users can easily perform statistical analysis or plot by themselves according to their preference. The third is .npy files, which contain more output information and intermediate result information such as the position of the activity of fly at every frame; thus, users can reload and reanalyze at any time. The fourth output is the config file related to user configurations, which can be used to modify or further develop in the future.

#### System Evaluation

To ensure the usage of different platforms and users, we evaluated the tracking accuracy rate of location and orientation (manually checked random frames of different videos) of EasyFlyTracker. Images of frames were randomly generated from three different videos taken at random (November 17, 2020, December 1, 2020, and December 4, 2020). For the location evaluation, we have used 100 random frames for each video. Each frame is a picture recording location of each fruit fly at the corresponding time point. Then, three different people manually judged the accuracy rate of tracking of each fruit fly. We distinguished the consistency of tracking location and location of fly in each randomly generated image and numbers of mistracked flies were recorded. Tracking errors were defined as those without recognizable location or where the cross was obviously not in the center of the fly. Finally, the average accuracy of location of three videos evaluated by three people was calculated as the accuracy of the tracking rate. For the orientation evaluation, we have used 600 random frames for each video and checked one fruit fly per frame. In total, three people manually checked the same 1,800 fruit flies and recorded three types of evaluation result including correct, wrong, and indistinguishable. After removing the indistinguishable cases, the average accuracy of orientation was then calculated.

### Other Information

Statistical analysis was performed using Python (version 3.8.3). The Kruskal–Wallis *H*-test (SciPy, version 1.5.0) was used for comparisons of groups. A value of *p* < 0.05 was considered to indicate statistical significance. The website was built mainly using VUE version 2.6 and Spring Boot version 2.4.0.

## Results and Discussion

### Overview of EasyFlyTracker

EasyFlyTracker is an open-source package based on video tracking to analyze locomotor and sleep activity of fruit fly that can be run interactively through a graphical user interface. The software can be easily customized to accept most of the common video formats such as MP4, AVI, MOV, FLV, and so on. It can track single adult individual flies in parallel and quantify their locomotor and sleep activity. The main function of EasyFlyTracker includes two aspects (Figure 2): (1) track the position of each fly and store it. Tracking is achieved through four steps: background modeling, foreground segmentation, coordinates calculation, and orientation estimation of *Drosophila*. The average tracking accuracy rate of location and orientation are 99.89 and 87.75% separately, which were manually evaluated random frames of different videos by three people (summary of tracking accuracy rate can be found in Supplementary Table 1) and (2) define, analyze, and visualize locomotor activity and sleep behaviors from various aspects including average distances every 10 min per fly (or other customized time interval), statistics of sleep status, statistics of average angle change, and the frequency of the fly passage at each position, and so on. It has been successfully installed and ran at the cross-platform level (Supplementary Table 2) by different person. More details on behavior definitions, outputs, and plot parameters can be found in the methods.

### Online Website and Usage of EasyFlyTracker

**FIGURE 3:**
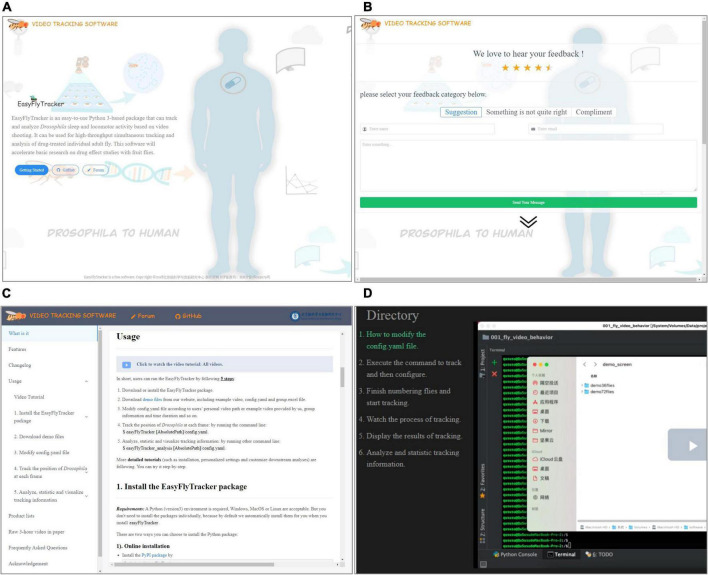
The website of EasyFlyTracker. **(A)** Home page. **(B)** Feedback page where users can add comments and suggestions for better upgrade interaction. **(C)** Detailed usage example. **(D)** Step-by-step video tutorials.

We provide the special website http://easyflytracker.cibr.ac.cn (home page, see Figure 3A) with feedback page (Figure 3B), where users can add comments and suggestions for better upgrade interaction and detailed usage example (Figure 3C) and step-by-step video tutorials (Figure 3D). In short, users can run EasyFlyTracker by the following steps: (1) download or install the EasyFlyTracker package; (2) download demo files from our website including example video, *config.yaml*, and group.excel file; (3) modify *config.yaml* file according to the personal video path of user or example video provided by us, group information and time duration, and so on; (4) track the position of *Drosophila* at each frame by running the command line: *easyFlyTracker config.yaml*; and (5) run other command lines to analyze and statistically track information: *easyFlyTracker_analysis config.yaml*. More detailed tutorials (such as installation, personalized settings, and customized downstream analyses) are available from our website. Technical comments and suggestions can also directly add to GitHub.^3^

### Psychiatric Drug Treatment Study of EasyFlyTracker

**FIGURE 4:**
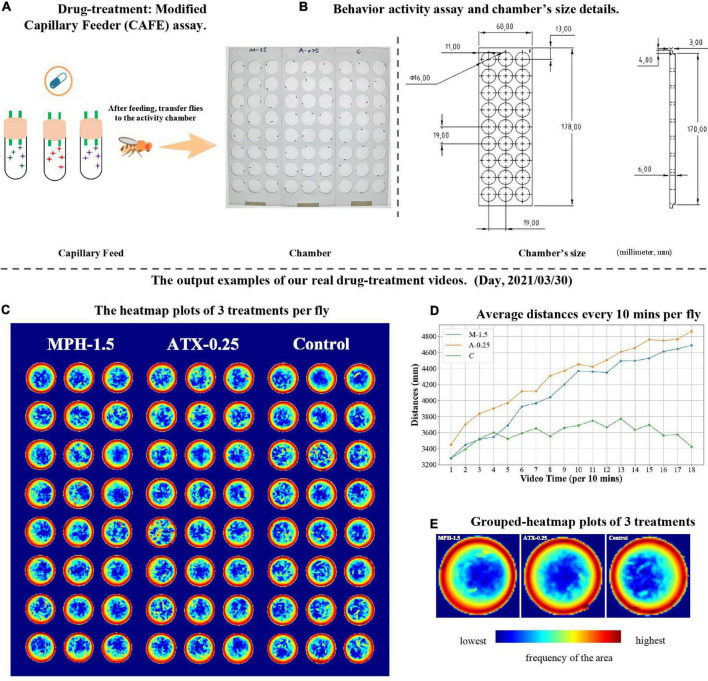
An example of psychiatric drug treatment study using EasyFlyTracker. **(A)** Drug treatment by modified capillary feeder (CAFE) assay. **(B)** Example of activity assay and size of chambers for the three treatments. Panels **(C–E)** plots are output examples of our real drug treatment video, which was taken on March 30, 2021. **(C)** Heatmap plot of the frequency of each fly. The relative frequency of the fly passage at each location was plotted (red indicates the highest frequency in the area; dark blue indicates that no flies ever transitioned through this position). **(D)** The average distance every 10 min per fly was significantly increased in the methylphenidate (MPH) (M-1.5)- or atomoxetine (ATX) (A-0.25)-exposed group compared to the control (C) group throughout the 3-h video. **(E)** Grouped-heatmap plots of three treatments. The color legend is the same as in **(C)**.

We applied EasyFlyTracker to 3-h videos recorded of *Drosophila* treated with wild-type (WT) *w*^1118^ control, MPH (a stimulant), and ATX (a non-stimulant) (Sigma-Aldrich, Shanghai, China). MPH and ATX are two commonly used drugs to treat ADHD symptoms of inattention, hyperactivity, and impulsivity in humans (Cortese, 2020). *Drosophila* breeding and modified capillary feeder (CAFE) assay (Diegelmann et al., 2017) for drug feeding (a cartoon example is shown in Figure 4A) can be found in Supplementary Material. After drug feeding, we placed one adult fruit fly in each arena and engaged in simultaneous tracking (24 flies per treatment and 72 flies in total, Figure 4B). Based on EasyFlyTracker, the locomotor activity of fruit fly was monitored by video and short-term distances were quantified. Our software reported that when WT male flies were exposed to MPH, the behavior of the flies produced hyperactivity-like behavior (higher locomotor activity) compared to controls (van der Voet et al., 2016; Rohde et al., 2019). We observed a significant increase in average distances over time per fly in MPH- (Kruskal–Wallis *H*-test: *p* = 1.93e-03) or ATX-exposed individuals (Kruskal–Wallis *H*-test: *p* = 4.48e-06) (Figure 4D), which is in agreement with published results (Rohde et al., 2019). Meanwhile, the corresponding average sleep time per 30 min was shown (Supplementary Figure 1), but no clear pattern observed during the length of the video. In addition, the heatmap plot of the frequency per fly (Figure 4C) showed the preferential status of each fly among our 3-h videos and grouped heatmaps of the three treatments were also provided (Figure 4E). They clearly show that flies moved continuously along the edges. This may be due to edge preference or repetitive stereotyped movements. We also provided angle change plot (Supplementary Figure 2) and movement plots of different treatments (Supplementary Figure 3) to help illustrate more details of the activities of fruit flies. It turned out that increased angle change activities associated with the treatment groups (Supplementary Figure 2) similar as that of moving activities (Figure 4D). It makes sense that angle change represents one form of routine activities. The above results indicate that EasyFlyTracker can help users to reveal the effects of adult *Drosophila* locomotor activity after drug treatment.

As a bonus, EasyFlyTracker can be easily transferred to other *Drosophila*-like animals or even other animal models such as worm and mouse, as we provide detailed tutorials and open-source code on the website.^4^ If users wish to extend to other animal models, we still recommend testing the accuracy of tracking first. In addition, this study has some limitations. We did not conduct a real-time tracking function of the software because during our development process, it was considered more important to prove the offline accuracy rather than real-time tracking and analysis. Also, in order to maintain an open development for better expansion by others, we provided all source code rather than developing it as a fixed-format program. Tracking of group behaviors was not considered in current version, since we have not figured out a solution at a low cost. Finally, our software is designed for adult fruit fly, thus we did not test its applicability to larval fruit fly. In the future, we will optimize and upgrade the software taking into account the above elements and incorporating user comments. In summary, we developed a Python package, called EasyFlyTracker, which is simple, stable, and reliable for analyzing the locomotor activity of fruit flies and it is easy to rebuilt equipment, which is suitable for the software. We hope that this system can achieve large-scale screening of drug response and even target genes in the future, thereby providing clues for psychiatric research and is expected to provide precision medicine research and new drug development models for its drug treatment in *Drosophila* as well as other animals.

## Data Availability Statement

The datasets generated for this study can be found in the article/Supplementary Material. Tutorials and open-source code are available at http://easyflytracker.cibr.ac.cn.

## Author Contributions

LiZ and SQ conceived the project and coordinated the collaboration. SQ designed the project, conducted drug and behavior experiments, designed the website, and drafted all the manuscripts. LiZ supervised the project plan. QZ wrote the program. HZ built the website. YG and YW fed fruit flies. YM purchased and setup the recording equipment. SQ and QZ mainly tested the function of the program. YG and ZW were involved in testing the accuracy and the installation. XS and LeZ made the industrial drawings of the activity chambers. QY provided the drugs. LK gave detailed suggestions on the software. SQ, QZ, LK, and LiZ revised the manuscript together. All authors contributed to the article and approved the submitted version of the manuscript.

## Conflict of Interest

The authors declare that the research was conducted in the absence of any commercial or financial relationships that could be construed as a potential conflict of interest.

## Publisher’s Note

All claims expressed in this article are solely those of the authors and do not necessarily represent those of their affiliated organizations, or those of the publisher, the editors and the reviewers. Any product that may be evaluated in this article, or claim that may be made by its manufacturer, is not guaranteed or endorsed by the publisher.

## Footnotes

1
https://www.python.org/

2
http://easyflytracker.cibr.ac.cn/#/document

3
https://github.com/azzhu/EasyFlyTracker/issues

4
http://easyflytracker.cibr.ac.cn
