# Supplementary material for: EasyFlyTracker: A Simple Video Tracking Python Package for Analyzing Adult Drosophila Locomotor and Sleep Activity to Facilitate Revealing the Effect of Psychiatric Drugs
Source: Front Behav Neurosci. 2022 Feb 10;15:809665. doi: 10.3389/fnbeh.2021.809665 (PMC8868375; doi:10.3389/fnbeh.2021.809665)
Supplement: Supplementary file 1 [file Data_Sheet_1.pdf]

Supplementary Materials of EasyFlyTracker

Drosophila breeding

Wild-type (WT) *w^{1118} Drosophila melanogaster* was obtained from the FangJing Company, and the population was maintained at 25°C under a 12 hr:12 hr light:dark photoperiod. All flies were reared on standard *Drosophila* medium (corn, sugar, yeast, agar) in a 25°C climate chamber at ~60% relative humidity with a 12 hr:12 hr light:dark cycle. Flies used for testing in all behavior experiments were 3- to 5-day-old adult males after eclosion at the time of behavioral activity assay experimentation.

Behavioral activity assay

Users can also prepare flies in other ways according to their experimental design. Here, we provided our activity assay details for informational purposes. The behavioral activity assay included two parts, as shown in **Figure 4**. First, flies were exposed to one of the three treatments (control, MPH or ATX) (Sigma–Aldrich China, Shanghai) using the modified capillary feeder (CAFE) assay (Diegelmann, et al., 2017) for approximately 24 hours for visual control of food intake **(Figure 4A)**. Five (~3 days old) males were transferred to feeding vials containing two 5-μl capillary tubes extending down into the vial. The feeding vials were topped with an oil layer and contained within a tightly sealed container with high humidity (~90%) to minimize evaporation. The control treatment consisted of 5% sucrose (SUC) and yeast solution (with 5% blue food dye); the methylphenidate (MPH) treatment was a 5% SUC and yeast solution (with 5% blue food dye) containing 1.5 mg ml^{-1} MPH; and the atomoxetine (ATX) treatment was a 5% SUC and yeast solution (with 5% blue food dye, Sigma–Aldrich China, Shanghai) containing 0.25 mg ml^{-1} ATX. Next, flies were relocated from the CAFE assay to the customized activity chambers. We placed one fly in one hole and engaged in simultaneous tracking (24 flies/treatment and 72 flies in total, **Figure 4B**). Each activity plate contained 72 or any customized circular arenas (chamber size is also shown in Figure 4B: diameter is 16 mm and height is 6 mm), which were placed on top of a light box and enclosed within a separate room to minimize external disturbance. After the flies adapted to the video environment (lights were turned on, usually from 09:00 to 10:00 am), the camera was used to start the video shooting for 3 hours (or other customized time), and then **our developed software EasyFlyTracker** was used to track and analyze the locomotor activities of the flies in different treatment groups through the saved videos.

Details of visualization parameters

Detailed information related to visualization parameters of our software is provided below.

The activity plots. The activity plots show the activity of different *Drosophila* groups during different time intervals. The horizontal (x) axis represents the different time intervals, which can be set by users through parameter file *config.yaml*, and the vertical (y) axis represents the total distances moved by each fruit fly on average during that time interval.

The statistics of sleep time. *Drosophila* that remains stationary for more than 5 minutes (consistent with published literature (Shaw, et al., 2000)) are considered to be sleeping. However, it is necessary to set a threshold to regulate the definition of stationarity. Even if the flies are truly stationary, slight changes in light and slight camera shaking at different times can cause the center of stationarity of the flies to not be exactly the same. Thus, the threshold is set to 1.5 mm per second in this software, which means that a fly is judged to be stationary if its center of repose changes by less than 5 pixels in a second.

The heatmap plots. The heatmap plots show the relative frequency of the fly passage at each position, and both frequency per flies and grouped heatmaps are provided. If necessary, sleep intervals can be removed from the heatmap plots with the "heatmap_remove_sleep" parameter defined in "*config.yaml*". The density of the flies at each arena position is calculated by the accumulation of all frame fly segmented regions. The histogram of the original values is not evenly distributed, and if converted to a pseudo-color map on an equal scale, many details are often lost. Thus, we used histogram equalization for the heatmap, which not only retains more details but also has a better viewing effect.

The angle change plots. The angle change plots show average angle change per second per fruit fly in a self-defined time interval, which is the sum of the angle change values from each frame to the previous frame. The whole-body orientation of the fruit fly is determined according to the method '**Orientation estimation of *Drosophila***' in the main text, and then angle changes between each frame is calculated based on orientation. Users could specify different time interval (default is 10 mins) to show the statistics of angle change during the flies' movement.

The regional preference of *Drosophila* movements. The graph is calculated from the heatmap of each *Drosophila* and more visually shows the regional bias of *Drosophila* movement. The red line with arrows indicates the direction of movement of the barycenter of the heat map relative to the center of the circle (regional bias of the fruit fly), and the size of the radius of the cyan circle represents the magnitude of the regional bias of the fly.

Table S1. Summary of tracking accuracy rate of EasyFlyTracker. Manually checked the tracking accuracy rate of different videos by three persons.

The tracking accuracy rate of location and orientation.

	**Day-2020/11/17**		**Day-2020/12/01**		**Day-2020/12/04**	
	Location	Orientation	Location	Orientation	Location	Orientation
1 (Yuan Gao)	99.90%	88.76%	99.96%	91.32%	99.97%	90.62%
2 (Qingjie Zhu)	99.93%	88.53%	99.92%	84.98%	99.94%	84.63%
3 (Susu Qu)	99.83%	89.23%	99.67%	86.60%	99.92%	85.05%
**Average**	99.89%	88.84%	99.85%	87.63%	99.94%	86.77%
**Average rate of 3 days**	**Average rate of location: 99.89%. Average rate of orientation: 87.75%**					

Table S2. Summary of usage information of different platforms. Three persons used different platforms to successfully install and track the same video.

The usage of different platforms by different users.

**Users**	**Platforms**		
	**Windows 10**	**MacOS**	**Linux**
**1 (ZhichengWang)**	Yes	/	/
**2 (QingjieZhu)**	/	/	Yes
**3 (SusuQu)**	/	Yes	/

Figure legends.

Figure S1. Average sleep time and proportion of sleep flies of each treatment. Per duration is 30 mins, and the whole video was 3 hours. The left y-axis shows the sleep time (min), and the right y-axis shows the proportion of sleep flies within the time interval. All sleep times were equal to the proportion of sleep flies multiplied by the fly number. This video was shot on 2021/03/30.

Figure S2. The angle change plot for the whole videos time. Statistics of average angle change per second per fruit fly of three treatments. The x-axis represents different time interval and the default is 10 mins, the y-axis shows the angle change value per second per fruit fly. This video was shot on 2021/03/30.

Figure S3. The regional preference of *Drosophila* movements of different treatments. The red line with arrows indicates the direction of movement of the barycenter of the heat map relative to the center of the circle (regional bias of the fruit fly), and the size of the radius of the cyan circle represents the magnitude of the regional bias of the fly. This video was shot on 2021/03/30.

References

Diegelmann, S., *et al.* The CApillary FEeder Assay Measures Food Intake in Drosophila melanogaster. *J Vis Exp* 2017(121).
