# Supplementary figures and images for: EasyFlyTracker: A Simple Video Tracking Python Package for Analyzing Adult Drosophila Locomotor and Sleep Activity to Facilitate Revealing the Effect of Psychiatric Drugs
Source: Front Behav Neurosci. 2022 Feb 10;15:809665. doi: 10.3389/fnbeh.2021.809665 (PMC8868375; doi:10.3389/fnbeh.2021.809665)

Average sleep time per flies per duration & Proportion of sleep flies

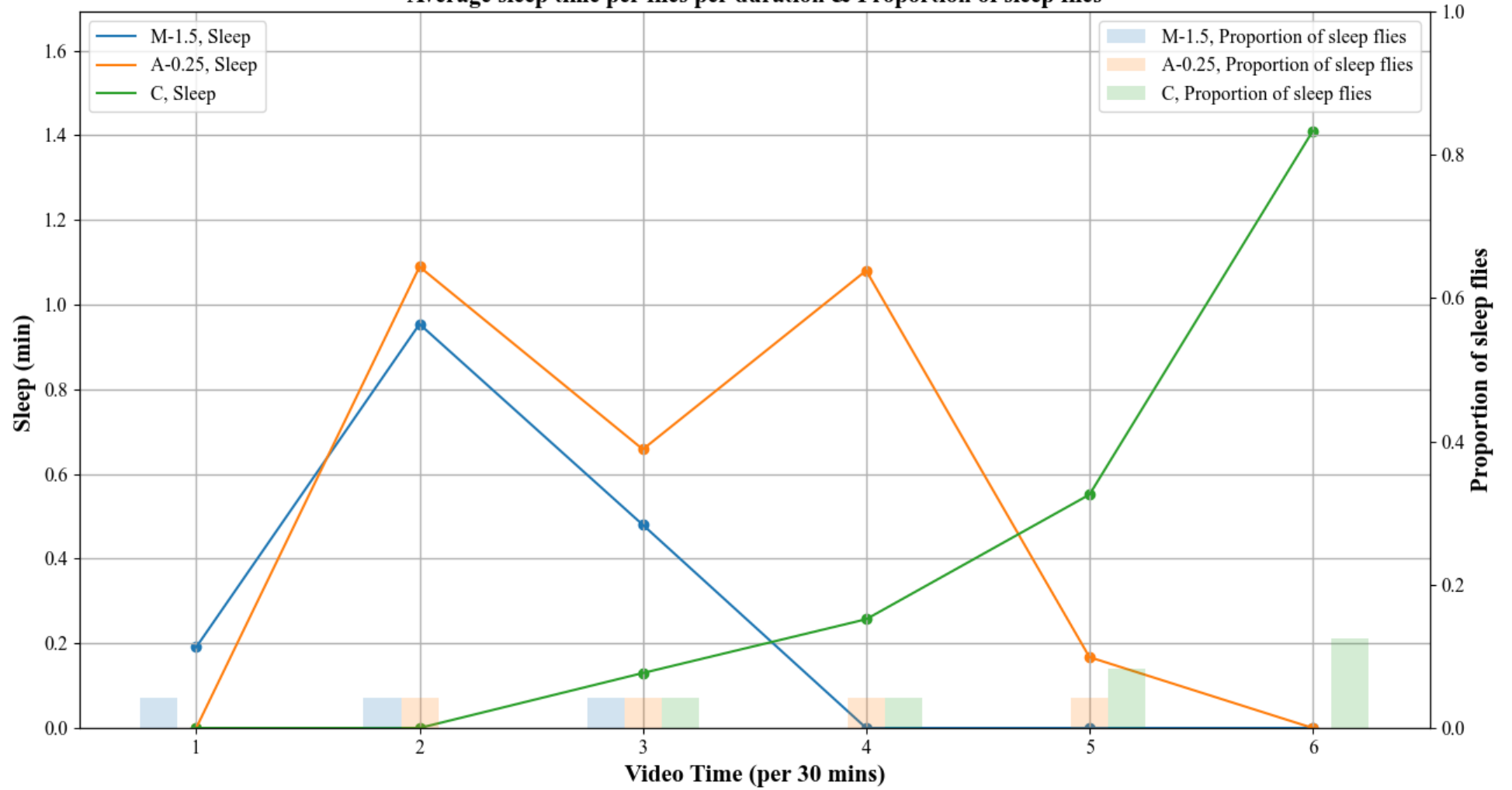

Supplement: Supplementary file 2 [file Image_1.pdf]

The angle change plot for the whole video time

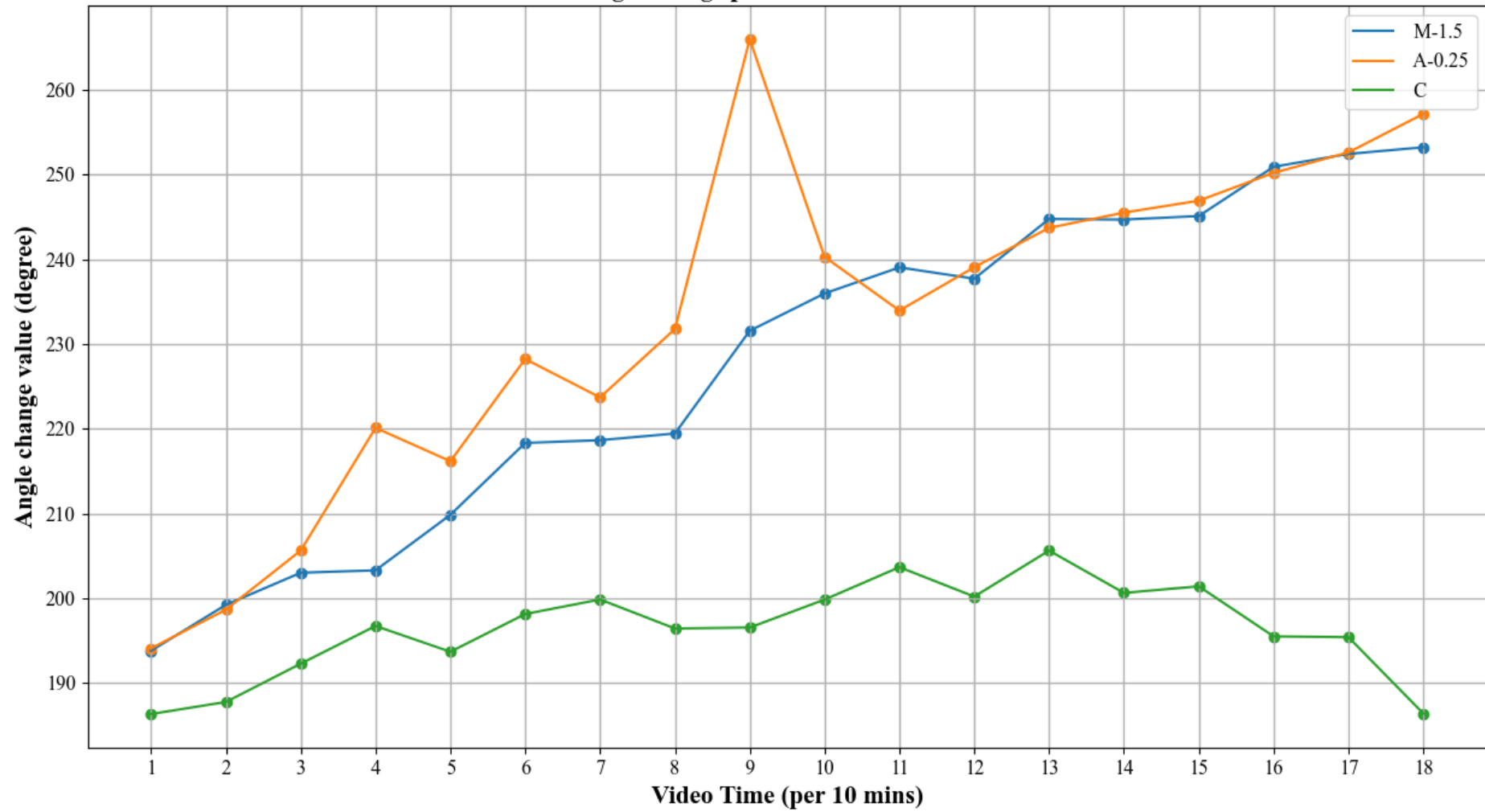

Supplement: Supplementary file 3 [file Image_2.pdf]

**MPH-1.5**

**ATX-0.25**

**Control**

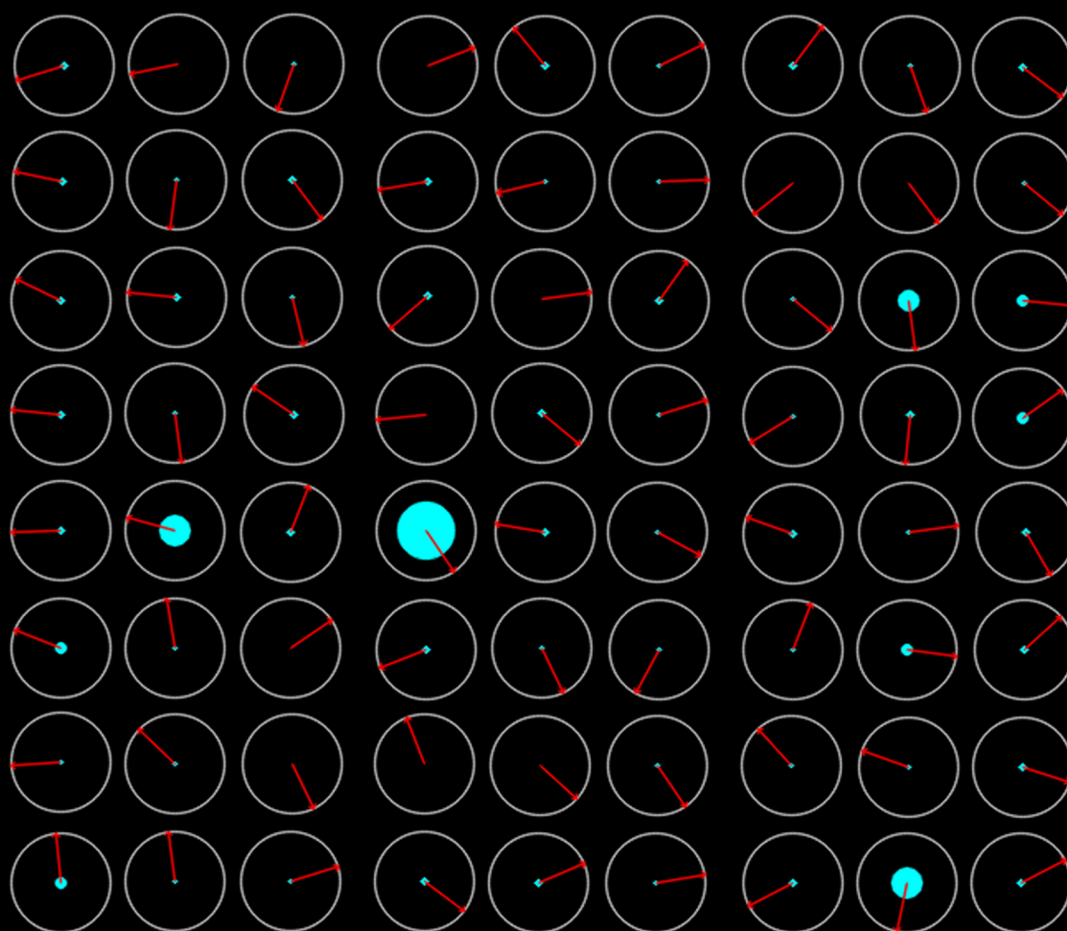

Supplement: Supplementary file 4 [file Image_3.pdf]
